# Angiography Revealing a Possible Paraneoplastic Vasculitis: A Case Report

**DOI:** 10.7759/cureus.63229

**Published:** 2024-06-26

**Authors:** Kristina McLeod-van Amstel, Elie Barakat

**Affiliations:** 1 Interventional Radiology, Edward Via College of Osteopathic Medicine, Monroe, USA

**Keywords:** small vessel vasculitis, anca associated vasculitis, breast oncology, clinical rheumatology, microscopic polyangeitis, ct angiogram, immune-mediated vasculitis, paraneoplastic syndromes

## Abstract

Various conditions under the umbrella term of vasculitis have been well documented in the literature. These have been classified into small, medium, and large vessel vasculitis. In addition, vasculitis has been categorized into radiation-induced, systemic, and paraneoplastic. Of these, paraneoplastic vasculitis accounts for 2-5% of all cases of vasculitides and is less well documented. We present a case of a female patient with a history of breast cancer presenting with an upper gastrointestinal tract (GI) bleed, which subsequently revealed an underlying diagnosis of systemic vasculitis, possibly paraneoplastic. This case highlights the importance of imaging for revealing underlying vasculitis as an etiology of GI bleed.

## Introduction

Vasculitis refers to a rare group of conditions affecting blood vessels. It is categorized into the following three subtypes: radiation-induced vasculitis, systemic vasculitis, and paraneoplastic vasculitis, which is even rarer and accounts for 2-5% of all cases of vasculitides [1]. Radiation-induced vasculitis has been found in patients undergoing radiotherapy and is diagnosable upon imaging [2]. The vasculitis-related changes in small- and medium-sized vessels manifest as “beading”, reflecting the narrowing of segments of the vessel and aneurysmal-like ballooning in other segments [3]. Radiation-induced vasculitis can be differentiated from systemic vasculitis based on the extent of change seen in imaging. If it is confined to the field of radiation, a diagnosis of radiation-induced vasculitis can be made [2]. However, findings outside of the field of radiation are suggestive of a systemic form of vasculitis.

Systemic vasculitis is often classified into large, medium, or small vessel vasculitis depending on the vessels that are affected the most [4]. While it is difficult to ascertain the specific type of vasculitis from imaging alone, many of the vasculitides have other clinical features and serology profiles that help delineate the type. Large vessel vasculitis conditions include giant cell arteritis and Takayasu's arteritis. These conditions classically present with ocular disturbances [4], with giant cell arteritis also presenting with a headache [5]. Takayasu's arteritis patients are often young to middle-aged with arteriosclerosis of the aorta and can also present with poorly palpable peripheral pulses [4].

Medium vessel vasculitis conditions include polyarteritis nodosa, which most commonly affects the kidneys, leading to renal failure or renal-associated hypertension, gastrointestinal (GI) tract involvement with abdominal pain/nausea/vomiting, and, less frequently, liver involvement [4]. In rare cases, GI involvement may manifest as hemorrhage or bowel ischemia leading to perforation. Serology for polyarteritis nodosa is unfortunately variable with only 30% of patients found to be positive for hepatitis B antigen and unreliable anti-neutrophilic cytoplasmic antibody (ANCA) titers making the diagnosis harder to confirm.

Meanwhile, a small vessel vasculitis, like microscopic polyangiitis, may present with a bleeding profile including the GI tract, hemoptysis, or hematuria with gastrointestinal perforation being a more severe complication [6]. Interestingly, in our case, tumor antigens were mentioned as a possible trigger for microscopic polyangiitis [4]. Other small vessel vasculitides include Wegener’s granulomatosis, Churg-Strauss syndrome, and cutaneous leukocytoclastic angiitis. Of these, Wegener’s granulomatosis, Churg-Strauss syndrome, and microscopic polyangiitis have been found to be predominantly positive for some type of ANCA [6]. However, it should be noted that not all patients with these small vessel vasculitide profiles are positive for ANCA.

Wegener’s granulomatosis involves the respiratory tract and kidneys with the rare involvement of the GI tract. In cases of GI involvement, patients present with an inflammatory bowel disease picture [4]. Cutaneous leukocytoclastic angiitis often has predominately cutaneous manifestations and may not be systemic in nature. Patients with Churg-Strauss syndrome experience three phases of symptoms including asthma, eosinophilia affecting the lungs or stomach manifesting as pneumonia or gastroenteritis, and granulomatous inflammation [4,6]. These patients also more frequently manifest neuropathy and cardiac involvement compared to other small vessel vasculitides [6]. Interestingly, the small vessel vasculitides have been more frequently implicated as paraneoplastic vasculitis [7].

Paraneoplastic vasculitis is defined as vasculitides occurring in conjunction, before, or after a malignancy [7]. Its presence can also suggest a recurrence of the primary malignancy. This has most commonly been associated with hematologic cancers [8] as a leukocytoplasmic vasculitis belonging to the small vessel vasculitis; however, a recent study involving 15 cases has reported vasculitis concurrent with solid malignancies as well [7]. One theory that has been proposed to explain its pathogenesis involves the release of immunogenic factors from the tumor forming immune complexes within the circulatory system leading to a vasculitis picture [1,9,10]. As more and more cases are documented, the following three criteria to establish the diagnosis of a true paraneoplastic vasculitis have been suggested: a close temporal relationship between the malignancy and vasculitis, a parallel course [1], and the resolution of the vasculitis upon treatment of the underlying malignancy [7].

## Case presentation

**Figure 1:**
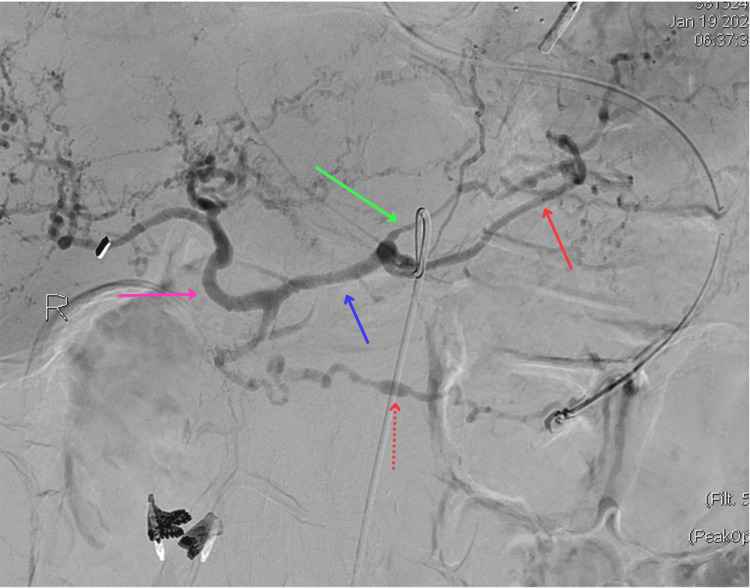
Angiogram of the celiac trunk The red solid arrow indicates the splenic artery with a noted area of saccular dilation as indicated by the red dotted arrow. The blue arrow indicates the common hepatic artery. The pink arrow indicates the proper hepatic artery dividing into left and right hepatic arteries. The green arrow indicates the left gastric artery

**Figure 2:**
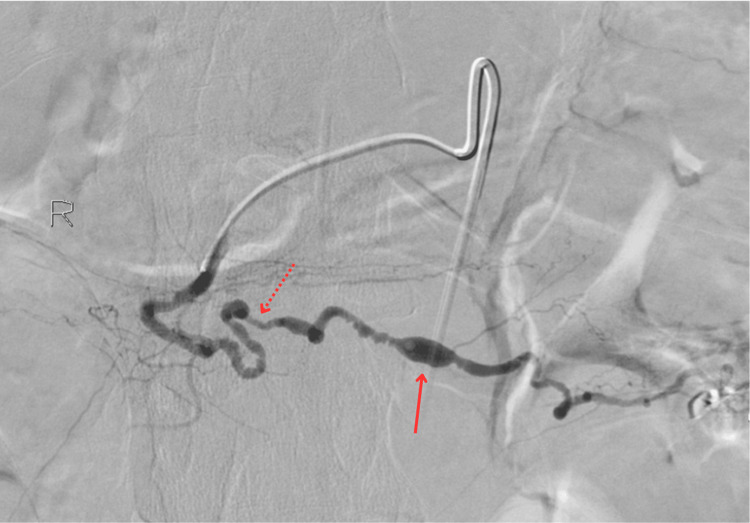
Angiogram of the distal gastroduodenum Angiogram of the distal gastroduodenum showing marked vascular wall irregularities of the right gastroepiploic arteries with intermittent narrowing and dilations. The solid red arrow points to significant saccular dilation. The dotted red arrow points to an area of significant stenosis

**Figure 3:**
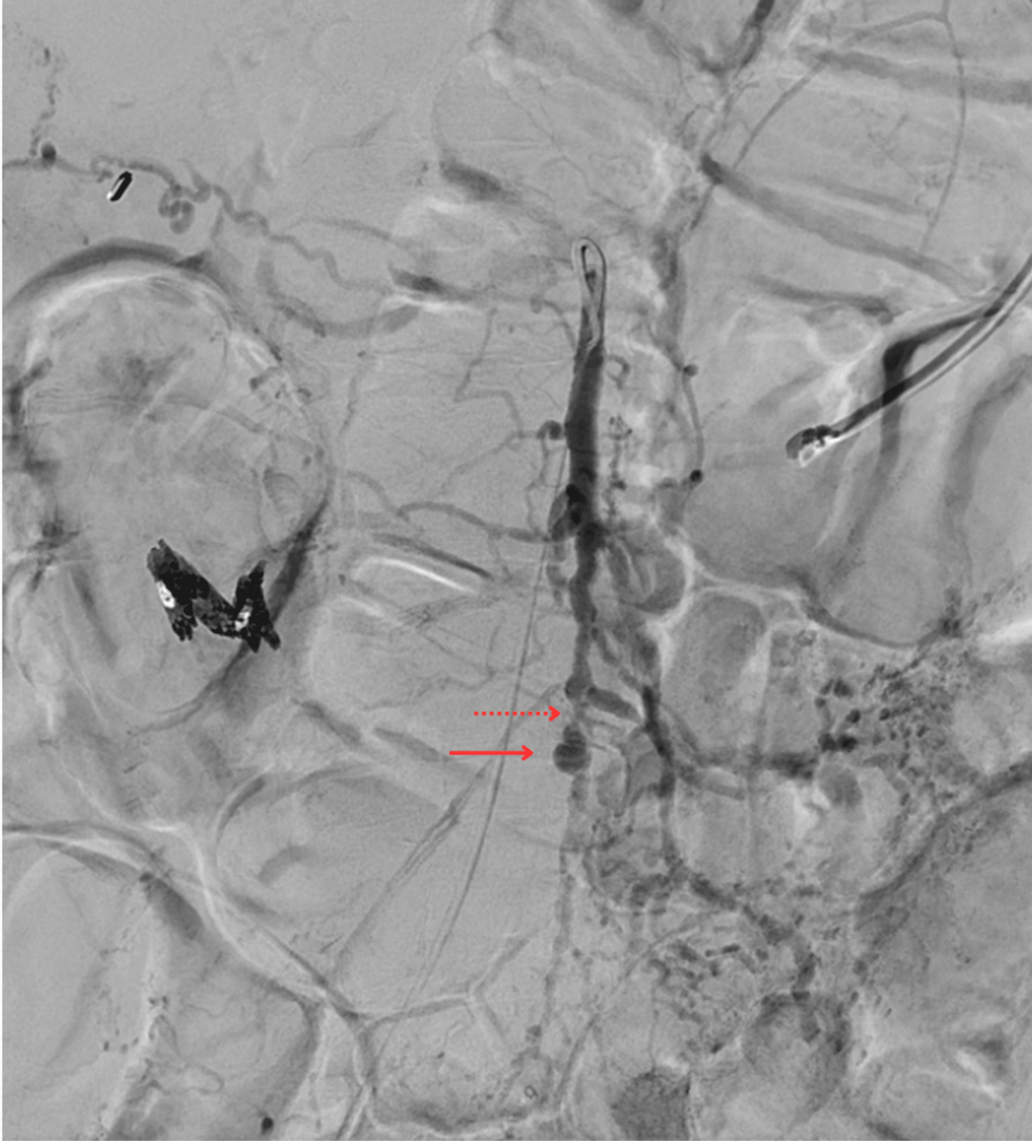
Superior mesenteric artery angiogram Superior mesenteric artery angiogram showing extensive vascular wall irregularities of the SMA and its branches with segmental stenoses and scattered aneurysmal dilations. One of the dilations is indicated by the solid arrow with one of the significant stenotic lesions indicated by the dotted arrow. The diffuse aspect of the vascular disease suggests vasculitis SMA: superior mesenteric artery

The patient was a 74-year-old Caucasian female with a medical history of stage IIIA right invasive mammary carcinoma (T2, N2, M0; ER + PR +) treated with bilateral mastectomy and chemotherapy in 2017. She presented to the emergency room with dark bloody vomitus and melena with a nondocumented history of gastric wall arteriovenous malformations. On physical exam and evaluation, the patient appeared to be in hemorrhagic shock. For the purpose of this case study on vasculitis, the physical exam ruled out any dermatological findings. Blood transfusions were initiated and endoscopy was performed to try to control the bleeding. However, the bleeding could not be controlled after multiple endoscopic attempts, and the patient was referred to IR for embolization. An angiogram was performed, which showed no active bleed; however, there were diffuse vascular irregularities of the hepatic arteries, splenic artery, gastroduodenal artery, and superior mesenteric artery and its branches (Figures 1-3).

**Figure 4:**
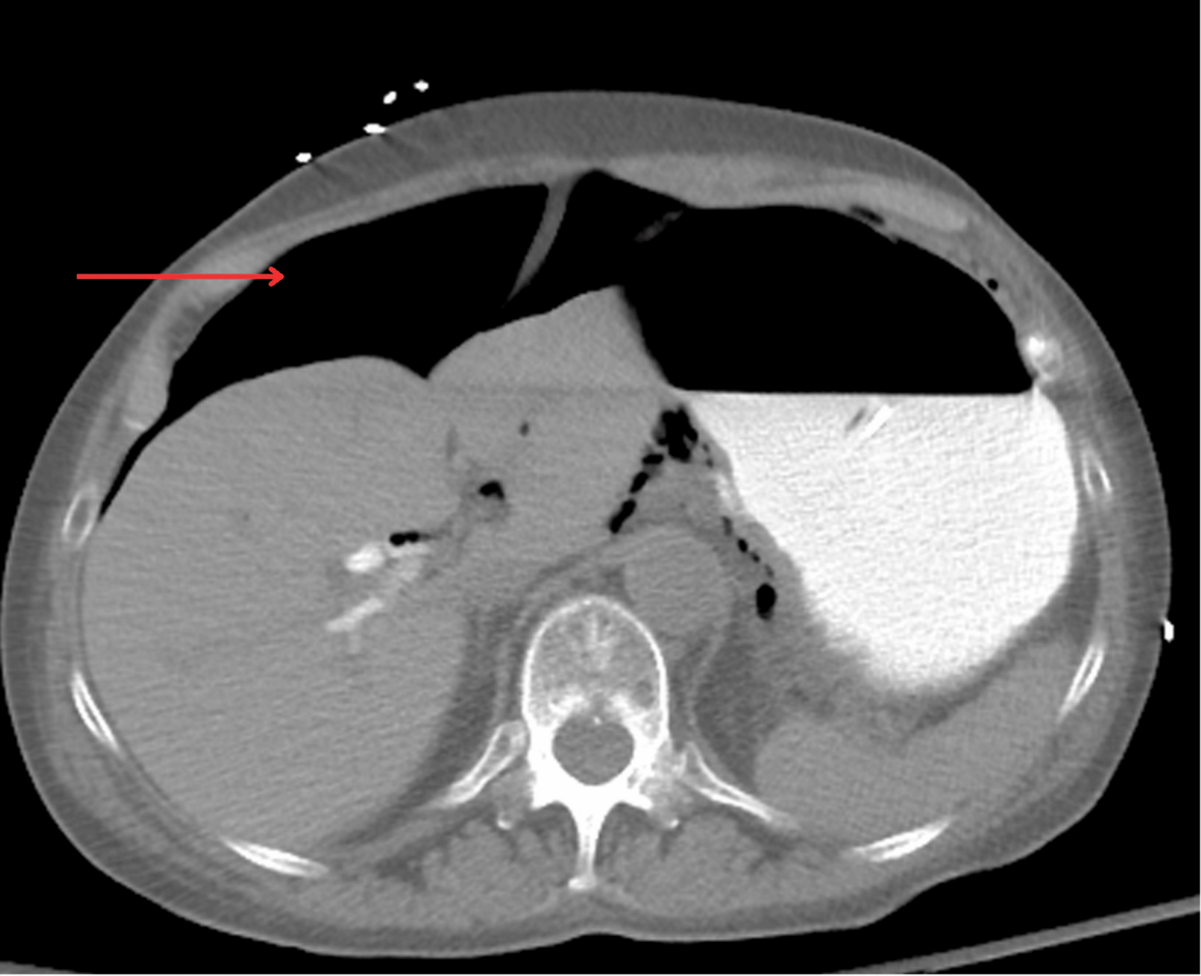
Abdominal CT showing pneumoperitoneum The red arrow indicates free air within the abdomen CT: computed tomography

The images obtained from the angiogram indicated an additional diagnosis of systemic vasculitis as the findings were not confined to the field of radiation, which was determined to be of low priority in terms of treatment order. The patient underwent empiric embolization of the gastroduodenal artery as the main source of bleeding was believed to be duodenal on esophagogastroduodenoscopy. Even though the embolization stopped the ongoing bleed, given the diffuse vascular disease, the patient was at high risk of bleeding from other sites. A CT scan of the abdomen and pelvis was also obtained, revealing free air within the abdomen suggestive of a perforation, likely iatrogenic secondary to multiple endoscopies, which was managed with surgical correction (Figure 4). The patient is currently recovering in the ICU and is still intubated. With the incidental finding of systemic vasculitis, a possible paraneoplastic vasculitis was also considered given her history of breast cancer; however, the treatment plan at this time is to continue monitoring the patient in ICU and ensure proper recovery. Investigations into the vasculitis have been deferred until the patient has the capacity to consent to further studies.

## Discussion

While the patient’s condition required extensive treatment not related to vasculitis, she was found to have vasculitis on imaging. The angiogram revealed extensive beading, multiple narrowing, and dilations consistent with vasculitis [3]. Vasculitis was determined to be systemic on imaging as it was not confined to the field of radiation [2]. Her history of breast cancer also hinted at a possible diagnosis of paraneoplastic vasculitis. Several cases of vasculitis linked with a diagnosis of cancer have been reported, supporting the evidence of paraneoplastic vasculitis [11]. This was considered even though a temporal relationship between her primary malignancy and vasculitis could not be established due to three factors. One, the extensiveness of her vasculitis suggests there could be a parallel course [1]. Secondly, it is possible that vasculitis appeared as a sign of recurrence of her malignancy as vasculitis may occur in conjunction, before, or after a malignancy [7]. Lastly, many patients may not recognize the initial signs of vasculitis to warrant a doctor’s visit, thereby leaving the vasculitis to progress until they present in an emergency condition as in this case.

In fact, it has been noted that many vasculitis patients present to the emergency department before a diagnosis of vasculitis [12]. Furthermore, a possible diagnosis of microscopic polyangiitis was considered, given the patient’s extensive GI bleed without cutaneous manifestations, ocular disturbances, renal or other upper respiratory tract involvement. This reasoning is similar to that proposed by Esperança-Martin et al. in the case of a patient with paraneoplastic vasculitis associated with breast cancer [1]. While further analysis of the vasculitis is required for confirmation, the patient is currently still in the ICU and intubated. Ideally, once the patient is stable, a consultation with a rheumatologist or hematologist and oncologist as well as further testing is needed to help identify the specific vasculitis. Specific laboratory tests would include an assessment of ANCA, antinuclear antibodies (ANA), hepatitis antibodies, CBC, biochemical profile, urinalysis, and urine electrolyte concentrations as well as a chest radiograph. In addition, further testing to identify a possible recurrence of breast cancer would be arranged as desired by the patient, which could help direct further management.

## Conclusions

While this case does not feature a confirmed paraneoplastic vasculitis, clinicians need to be aware of the condition as it can hint at a possible recurrence of malignancy. Additionally, it warrants further treatment and management otherwise not identified. Hence, this case demonstrates the importance of imaging in the diagnosis of systemic vasculitis as it had not been diagnosed prior to angiography.
